# The overexpression of R-spondin 3 affects hair morphogenesis and hair development along with the formation and maturation of the hair follicle stem cells

**DOI:** 10.3389/fphys.2024.1424077

**Published:** 2024-09-16

**Authors:** Alicja Olczak, Tomasz D. Pieczonka, Szymon Ławicki, Konrad Łukaszyk, Anna Pulawska-Czub, Linda Cambier, Krzysztof Kobielak

**Affiliations:** ^1^ Centre of New Technologies (CeNT), University of Warsaw (UW), Warsaw, Poland; ^2^ The Vision Center and The Saban Research Institute, Children’s Hospital Los Angeles, Los Angeles, CA, United States

**Keywords:** hair morphogenesis, hair development, hair cycle regeneration, hair follicle stem cells (HFSCs), R-spondin 3 (Rspo3), Wnt signaling

## Abstract

Mice hair follicles (HFs) are a valuable model for studying various aspects of hair biology, including morphogenesis, development, and regeneration due to their easily observable phenotype and genetic manipulability. The initiation and progression of hair follicle morphogenesis, as well as the hair follicle cycle, are regulated by various signaling pathways, of which the main role is played by the Wingless-type MMTV integration site family (Wnt) and the Bone Morphogenic Protein (BMP). During the hair follicle cycle, the BMP pathway maintains hair follicle stem cells (HFSCs) in a dormant state while the Wnt pathway activates them for hair growth. Given the pivotal role of the Wnt pathway in hair biology and HFSCs regulation, we investigated the influence of the Wnt modulator - R-spondin 3 (Rspo3), in these processes. For this purpose, we developed a transgenic mice model with the overexpression of *Rspo3* (Rspo3GOF) in the whole ectoderm and its derivatives, starting from early morphogenesis. Rspo3GOF mice exhibited a distinct phenotype with sparse hair and visible bald areas, caused by reduced proliferation and increased apoptosis of hair matrix progenitor cells, which resulted in a premature anagen-to-catagen transition with a shortened growth phase and decreased overall length of all hair types. In addition, Rspo3GOF promoted induction of auchene and awl, canonical Wnt-dependent hair type during morphogenesis, but the overall hair amount remained reduced. We also discovered a delay in the pre-bulge formation during morphogenesis and prolonged immaturity of the HFSC population in the bulge region postnatally, which further impaired proper hair regeneration throughout the mice’s lifespan. Our data supported that *Rspo3* function observed in our model works in HFSCs’ formation of pre-bulge during morphogenesis via enhancing activation of the canonical Wnt pathway, whereas in contrast, in the postnatal immature bulge, activation of canonical Wnt signaling was attenuated. *In vitro* studies on keratinocytes revealed changes in proliferation, migration, and colony formation, highlighting the inhibitory effect of constitutive overexpression of *Rspo3* on these cellular processes. Our research provides novel insights into the role of *Rspo3* in the regulation of hair morphogenesis and development, along with the formation and maturation of the HFSCs, which affect hair regeneration.

## Introduction

Mice hair follicles are very well-organized mini-organs, that are an exceptional model for delving into stem cell biology and exploring aspects of hair morphogenesis, development, and the regenerative hair cycle. This model is particularly advantageous due to ease of phenotype observation, genetic manipulability, and the ability to synchronize hair regeneration cycles, encompassing phases of growth (anagen), degeneration (catagen), quiescence (telogen), and exogen resulting in hair loss (Müller-Röver et al., 2001; Alonso and Fuchs, 2006).

During embryogenesis, HFs are formed through a series of epidermal-mesenchymal interactions, leading to the development and differentiation of epithelial down-growths into mature HFs (Hardy, 1992; Millar, 2002; Sengel and Mauger, 1976). The initiation and progression of HF morphogenesis, as well as the regulation of the regenerative hair cycle, are tightly controlled by two signaling pathways, Wnt and BMP. Both Wnt and BMP pathways play a crucial role in the development of mouse skin epithelium, which originates from the ectoderm, whereas Wnt/β-catenin signaling alone conditions the dermal fate around stage E10.5, emphasizing its key role in hair formation (Kobielak et al., 2003; Fuchs, 2007; Atit et al., 2006; Zhang et al., 2009; Plikus et al., 2008; Botchkarev et al., 2001; Gat et al., 1998). The canonical Wnt pathway is crucial for the initiation of hair morphogenesis as it induces the growth of placodes (Schneider et al., 2009). Previous studies have demonstrated that the absence of epidermal β-catenin expression and ectopic epithelial expression of the secreted Wnt inhibitor, Dickkopf 1 (Dkk1), results in failure of hair placode formation (Huelsken et al., 2001; Andl et al., 2002).

In the bulge region of HF resides a population of hair follicle stem cells, that is crucial for self-renewal and hair regeneration (Cotsarelis et al., 1990; Tumbar et al., 2004). Before the morphological establishment of the mature bulge by the end of the first postnatal telogen at P18, a pre-bulge region with label-retaining, slow-cycling cells (LRCs) is specified and formed during early HF morphogenesis, and it expresses early HFSCs markers, such as Lhx2, Sox9, Tcf3, NFATc1, but not yet cell surface marker CD34 (Kandyba et al., 2014; Vidal et al., 2005; Nguyen et al., 2006; Nowak et al., 2008; Horsley et al., 2008; Rhee et al., 2006). When maturation of bulge is completed at P18, HFSCs begin to express CD34, which together with α6-integrin constitute the only surface markers that can be used to purely isolate these stem cells alive from the whole skin by fluorescence-activated cell sorting (FACS) from wild mice (Trempus et al., 2003; Blanpain et al., 2004). In addition, mature bulge HFSCs also express high levels of keratin 15 (K15) (Liu et al., 2003; Morris et al., 2004).

During the growth phase, HFSCs undergo activation, primarily in the hair germ (HG) and subsequently in the bulge, contributing to the formation of a hair-bulb matrix (Greco et al., 2009). This matrix consists of transit-amplifying cells, which differentiate into the hair shaft (HS) and the inner root sheath (IRS) (Greco et al., 2009). After HF and epidermal morphogenesis are completed, keratinocytes located in the interfollicular epidermis (IFE) and the hair bulge play distinct roles in cellular fate determination (Levy et al., 2005; Leung et al., 2013; Nowak et al., 2008).

Rspo3 belongs to the R-spondins (Rspos) family, which are responsible for the activation and enhancement of Wnt signaling, thus participating in tissue development and homeostasis (Nagano, 2019). Rspos inactivate E3 ubiquitin ligases - Zinc And Ring Finger 3 (ZNRF3) and Ring Finger Protein 43 (RNF43) that control the rotation of Wnt-Frizzled receptor (Zebisch et al., 2013; Hao et al., 2012). They may act indirectly by binding to the leucine-rich repeat-containing G-protein-coupled receptors (LGR) 4-6, preventing degradation of β-catenin, which results in prolonged Wnt activation (Carmon et al., 2011; Carmon et al., 2012; de Lau et al., 2011; de Lau et al., 2014; Ruffner et al., 2012).

Rspos are a part of the inner-compartment mesenchymal signaling during the initiation of hair growth. They are secreted from the dermal papilla (DP) to synchronously stimulate the proliferation of HFSCs and epithelial progenitors through the canonical Wnt pathway, allowing for coordinated anagen entry of HFs (Raslan and Yoon, 2019). Although Rspo3 is a well-known factor in the development of many different tissues, its role and expression in adult skin and its appendages still require more insight (Raslan and Yoon, 2019; Hagner et al., 2020; Nagano, 2019). Until now, in the hair of adult mice, expression of *Rspo3* mRNA has been shown in the interface between HG and DP, upper bulge, lateral HG, mid bulge, lower isthmus, and IFE (Tsutsui et al., 2021).

Most studies have focused on revealing the role of Rspos in adult HF, demonstrating their activation of the proliferation of HFSCs and epithelial progenitors (Hagner et al., 2020; Chen et al., 2023; Li et al., 2016; Smith et al., 2016). Much less is known about the role of Rspos in hair morphogenesis and development. Taking into account previous studies showing the critical role of the Wnt pathway in hair morphogenesis and development, as well as the fact that Rspo3 can enhance activation of the Wnt signaling pathway, we hypothesized that Rspo3 plays an important role in the regulation of HFs morphogenesis, development, and regenerative cycle.

Therefore, this study aims to elucidate the role of R-spondin 3 in hair follicle morphogenesis, development, and the regenerative hair cycle. Specifically, we hypothesize that Rspo3 significantly influences these processes through its interaction with the Wnt signaling pathway. To investigate this, we utilized a transgenic mouse model with constitutive overexpression of Rspo3 in the entire ectoderm and its derivatives. This model allowed us to observe the phenotypic and molecular changes resulting from Rspo3 overexpression, shedding light on its impact on hair follicle structure, stem cell marker expression, and overall hair regeneration dynamics.

## Materials and methods

### Generation, genotyping and tissue collection

All mice were housed and bred within the animal facility at the Central Laboratory of Experimental Animals at The Medical University of Warsaw and the animal facility at the University of Warsaw. All the procedures were performed with the approval of the First Local Ethics Committee (permit no 215/2017, 495/2017 and 971/2020). To generate Rspo3GOF offspring, a series of matings were carried out using: Keratin 14 (K14) promotor-driven Cre recombinase (K14Cre) mice obtained from Jackson Laboratories (Vasioukhin et al., 1999), mice expressing Rspo3 gene in which chloramphenicol acetyltransferase gene cassette (CAT) flanked by LoxP sites is driven by the globally expressed chicken β-actin promoter (CAG) (Cambier et al., 2014), and mice having a Cre recombinase-dependent YFP reporter, inserted into the globally expressed Rosa26 locus together with a STOP cassette, flanked by LoxP sequences (Srinivas et al., 2001). As a control line (Con), K14Cre mice were used. All animals were genotyped using either ear or tail snips.

Whole back skin samples were collected, fixed in 4% paraformaldehyde for 2 h at room temperature, incubated in 30% sucrose overnight at 4°C, and frozen using Surgipath^®^ FSC 22^®^ Frozen Section Embedding Medium (Leica, 3801481). Skin samples were cut on Leica CM1860 cryostat to a thickness of 12 µm and stored at - 80°C.

### Protein isolation and Western blotting

Lysates from whole skin and keratinocytes obtained from P7 Rspo3GOF and Con mice were used for this analysis. Samples were lysed with Radio-Immunoprecipitation Assay buffer (RIPA; 150 mM sodium chloride, 1% Triton X-100, 0.5% sodium deoxycholate, 0.1% SDS, 50 mM Tris, pH 8.0) with protease inhibitors. Samples were briefly vortexed and incubated on ice for 20 min, and then centrifuged at 14,000 g for 15 min at 4°C. The supernatant containing proteins was collected and separated on 10% mini-PROTEAN TGX™ Precast Protein Gels, 12-well (Bio-Rad #4561035), and transferred onto a nitrocellulose membrane using a semi-dry Bio-Rad transfer system for 25 min at 12 V. Membranes were blocked in EveryBlot Blocking Buffer (Bio-Rad #12010020) for 8 min at room temperature. The membrane was cut at 75 kDa band, each part was incubated with appropriate primary antibody overnight at 4°C on a gentle shaker. Primary antibodies were diluted as follows: R-spondin 3 (1:2,000; R&D Systems #MAB41201), Vinculin (1:10,000; Abcam #ab129002). Membranes were washed in TBS-T and incubated with secondary antibodies conjugated with horseradish peroxidase diluted in EveryBlot Blocking Buffer (1/10,000), for 1 h at room temperature, on a gentle shaker. The enzymatic reaction was performed using Clarity Western ECL Luminol/enhancer + peroxidase solution substrate (Bio-Rad, #1705060). Amersham Imager 600 RGB was used to visualize the results.

### Reanalysis of scRNA-seq

The raw 10X sequencing data from the wild-type E13.5, E14.5, and E15.5 mouse skin samples deposited at GEO: GSE198487 (Qu et al., 2022) was processed with the standard 10X CellRanger pipeline. nUMI count matrices were obtained from fastq files through mapping to the mm10 reference genome. Then, they were filtered, centered, and normalized in Seurat (Macosko et al., 2015; Butler et al., 2018). scRNA-seq data were visualized with ggplot2 and cowplot R libraries.

### Hematoxylin and eosin staining and analysis

Cryosections of Rspo3GOF and Con mice skin were allowed to dry, and then washed in phosphate-buffered saline (PBS). Sections were incubated in hematoxylin for 2 min, then washed in distilled water and left to dry. Subsequently, sections were incubated in an eosin mix for 1 min and washed in 95% ethanol. The dry sections were mounted in 80% glycerol. Images were made using an inverted phase contrast microscope Eclipse TS100 (Nikon, Japan). Approximately 10 images from both Con and Rspo3GOF mice (*n* = 3) were used for the analysis of hair amount and length by the ImageJ program.

### Hair types, length, and amount

Hair from the neck region of P18 Rspo3GOF and Con mice (*n* = 3) were plucked and assessed under a dissection microscope (Leica MZ16 FA, Germany). Hair were differentiated based on type (guard, awl, auchene, and zigzag), counted, and measured. For analysis, 100 hair were taken from each mouse.

### Immunostaining procedure

Cryosections were briefly washed in PBS and left to dry. Subsequently, sections were blocked in a buffer containing 5% NGS, 1% BSA, and 0.2% Triton X-100 in PBS for 1 h at room temperature. Next, sections were incubated with primary antibodies overnight at 4°C. Antibodies were diluted in blocking buffer at the following dilutions: β-catenin (1:300; Sigma-Aldrich #C2206), Caspase-3 (1:100; Abcam #ab13847), CD34 (1:200; Abcam #81289), Cytokeratin10 (1:200; Abcam #76318), Cytokeratin14 (1:200; Abcam #181595), GFP (1:200; Abcam #13970), Ki67 (1:200; Abcam #ab15580), K15 (1:200; Invitrogen #PA599461), Lhx2 (1:200; Abcam #184337), Loricrin (1:300; Gifted by C. Jamora (Jamora et al., 2005)), Sox9 (1:200; Abcam #185966), Phospho–Smad1/5/9 (1:800; Cell Signaling Technology #13820), Phospo–Smad1/5 (1:800; Cell Signaling Technology #9516). Then, sections were washed in PBS and incubated with secondary antibodies diluted at 1:300 in blocking buffer for 1 h at room temperature in the dark. Sections were thoroughly washed in PBS, counterstained with a fluoromount containing 4′,6-diamidino-2-phenylindole (DAPI), and closed with cover slides. Prepared slides were stored at 4°C. Images were captured using a Zeiss LSM 700 confocal microscope or Zeiss Axis Observer fluorescent microscope.

### Oil Red staining

Cryosections of Rspo3GOF and Con mice skin were allowed to dry, and then washed in PBS. Subsequently, slides were incubated in 60% isopropanol for 5 min, and the in 0.5% Oil Red Solution for 15 min in room temperature. Therefore, sections were washed in double-distilled water and stained with Hematoxylin for 1 min. At final step, sections were washed in water and left to dry. Dried sections were mounted using 10% glycerol, and closed with cover slides. Images were made using an inverted phase contrast microscope Eclipse TS100 (Nikon, Japan).

### Keratinocyte isolation and *in vitro* culture

Keratinocytes were isolated from Rspo3GOF and Con mice at P7, following the protocol outlined in (Lichti et al., 2008). *In vitro* cell cultures were maintained in an incubator with a temperature 37°C, 5% CO_2_ concentration, and controlled humidity. Initially, keratinocytes were cultured on a mitomycin C -treated 3T3 fibroblast feeder layer in E-media (Rheinwald and Green, 1977) supplemented with 15% fetal bovine serum (FBS; HyClone, #SH 30071.03) and 0.3 mM Ca^2+^ (CaCl_2_, Chempur, #118748709) (E-MC). After the third passage, keratinocytes were cultured in E-media (Rheinwald and Green, 1977) supplemented with 15% FBS and 0.05 mM Ca^2+^ (E-LC), without a supporting layer of 3T3 fibroblasts, as described in (Kandyba et al., 2013).

### RNA isolation and RT-qPCR

Total RNAs were purified from keratinocytes using a RNeasy Mini Kit (Qiagen, #74104) according to the manufacturer’s instructions. The concentration and purity of isolated RNA were verified using a DeNovix DS-11 spectrophotometer. For reverse transcription, the High-Capacity cDNA Reverse Transcription Kit (Applied Biosystems™, #4368814) was utilized, using 2 μg of RNA as a starting material. The RT-qPCR reaction was performed using PowerUp™ SYBR™ Green Master Mix Kit (Applied Biosystems™, #A25742) on the Light Cycler 480 II (Roche). Samples were analyzed in triplicates using the 2^−ΔΔct^ method.

### Colony formation assay

A total of 2,000 cells from Con and Rspo3GOF (*n* = 3) were seeded into each well of a 6-well plate and cultured in the E-LC medium. The cell culture medium was replaced every 2–3 days. Cultures were terminated after 9 days, and cells were fixed with cold 100% methanol for 10 min followed by 4% PFA for 15 min at room temperature. Cell colonies were stained with 0.01% crystal violet solution. The results were visualized with Bio-Rad Molecular Imager GelDock XR+ and the colony size, as well as colony number, were counted using ImageJ software.

### Wound healing *in vitro* assay

Con and Rspo3 keratinocytes were cultured *in vitro* until they reached confluence. Subsequently, they were incubated with mitomycin C (final solution 8 μg/mL) for 2 h. After incubation, the medium was removed, and using sterile 10 μL pipette tips, two perpendicular scratches were made across the plates. Images of cell cultures were captured at 24-h intervals for 4 days or until the cells completely covered the scratches. ImageJ software was utilized for further analysis of the captured images.

### Fluorescent activated cell sorting (FACS)

The dorsal skin from one-year-old Con and Rspo3GOF mice was removed, and subcutaneous connective tissue and fat were scraped off using a scalpel. The skin was then digested with 0.25% trypsin (Biowest, #L0931-100) overnight at 4°C. To obtain a homogeneous cell suspension, the top skin layer was scraped off with a scalpel, and E-LC medium was added and pipetted. The suspension was filtered through a nylon cell filter with a mesh diameter of 70 µm and centrifuged for 30 min at 4°C and a speed of 300 RCF. The supernatant was then poured off, and the cell pellet was collected and suspended in 10 mL of cold PBS buffer with 1% Fetal Bovine Serum (FBS) without calcium ions. The suspension was filtered through a nylon filter with a mesh diameter of 40 µm and centrifuged again under the same parameters. The resulting cell pellet was suspended in a cold PBS buffer with 1% FBS without calcium ions, and cells were stained. Antibodies directed against the surface markers of HFSCs-CD34 (1:50; Invitrogen #56-0341-82) and α6-integrin (1:200; BD Pharmigen #555736), conjugated with appropriate fluorochromes were added to the cell suspension and incubated for 30 min at 4°C in the dark, with vortexing every 10 min. The cell suspension was washed three times with PBS buffer and centrifuged. Additionally, different controls were prepared: cells stained with single antibodies, an unstained control with endogenous EGFP/EYFP fluorescence, and a control showing no fluorescence. Immediately, before sorting, DAPI buffer at a concentration of 1:10,000 was added to the cell suspension to assess cell viability. The suspension was filtered through a nylon filter with a mesh diameter of 20 μm, and the desired cell population was sorted (Nowak and Fuchs, 2009). Sorting was performed on a FACSAria Fusion instrument (Becton Dickinson). The purity of the sort was checked by reanalysis. Changes in the cell population between Con and Rspo3GOF were analyzed using the FlowJo program.

### Statistical analysis

Statistical analyses were carried out using the Prism software package (GraphPad). The significance of differences between two groups was determined using the unpaired, two-tailed Student’s t test. Analyses of multiple groups were performed using One- Way ANOVA. Statistical significance was denoted by asterisks (P < 0.05 [*], P < 0.01 [**], and P < 0.0001 [***].The data are presented as mean ± SEM.

## Results

### Generation of mice with constitutive overexpression of Rspo3 in the epidermis and its appendages (Rspo3GOF)

**FIGURE 1:**
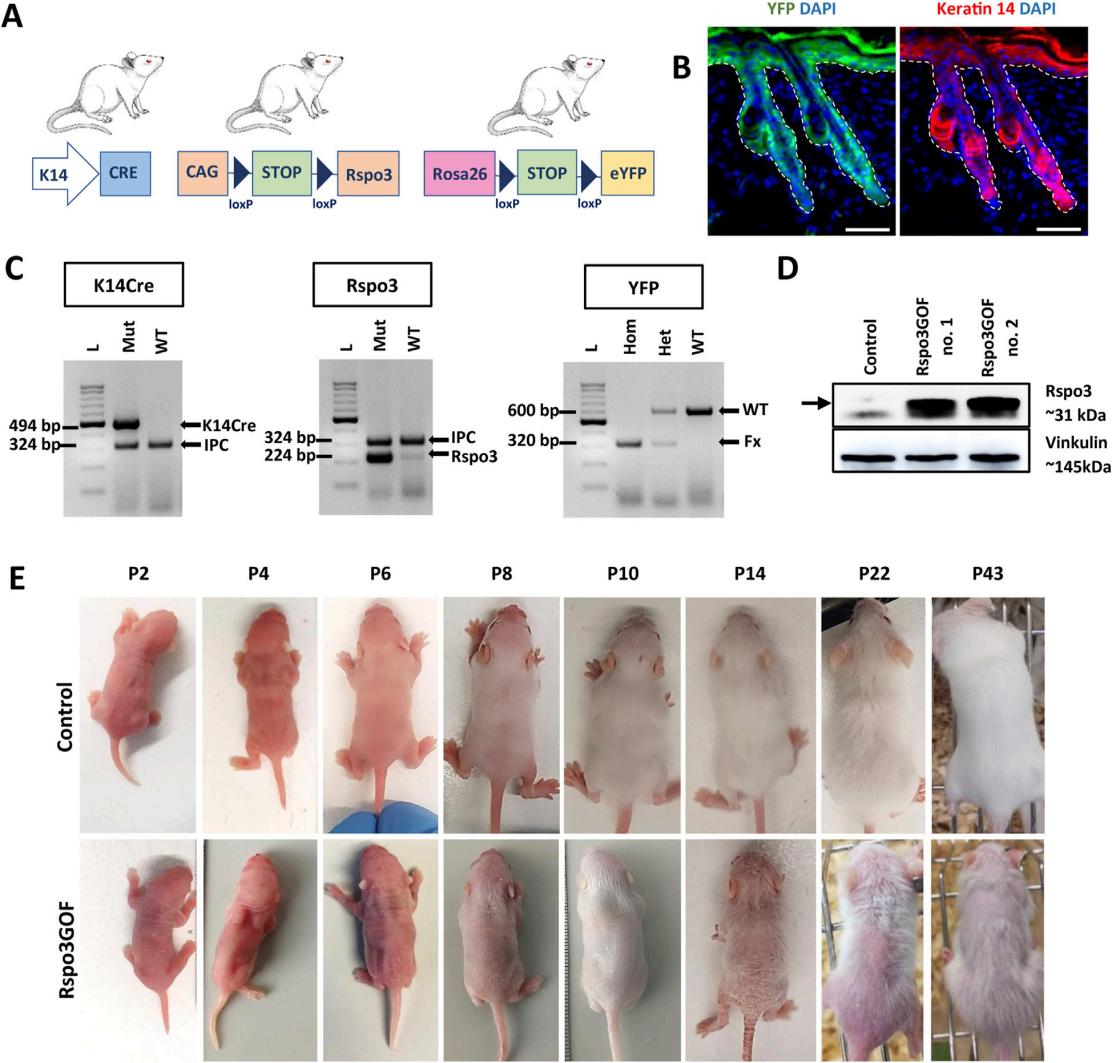
Generation of transgenic mice with constitutive overexpression of Rspo3 (Rspo3GOF) in the epidermis and its appendages. **(A)** Schematic representation of mating leading to generation of Rspo3GOF mice. K14Cre mice were crossed with CAG-lox-STOP-lox-Rspo3 transgenic mice, and Rosa26-lox-STOP-lox-eYFP reporter mice to generate mice with Cre-driven overexpression of Rspo3 in K14-positive YFP-labeled epidermal keratinocytes. Abbreviations: CAG, chicken β actin; YFP, yellow fluorescent protein; K14, keratin 14. **(B)** Immunofluorescence staining of Rspo3GOF back skin at P18, showing the expression pattern of YFP and Keratin-14. DAPI counterstaining was used to label cell nuclei. Scale bar = 50 µm. **(C)** Genotyping results confirmed the presence of the specific PCR products for K14Cre, Rspo3, and YFP genes in the transgenic mice. Arrows depict PCR products: K14Cre–494 bp., Rspo3–224 bp., YFP WT–600 bp., YFP flox/flox–320 bp., IPC–324 bp. Abbreviations: IPC, internal PCR control; WT, wild type. **(D)** Western blot results from whole skin lysates with Rspo3 antibody confirmed expected 31 kDa size bands recognizing Rspo3 overexpression in two biologically independent, genotypically positive Rspo3GOF transgenic mice. **(E)** The phenotype of Rspo3GOF and control mice during different postnatal developmental stages at P2, P4, P6, P8, P10, P14, P22, P43. Rspo3GOF mice demonstrate a strong phenotype with a very sparse hair coat and epidermis peeling.

To investigate the role of the Wnt pathway modulator, Rspo3, in hair morphogenesis, development, and regeneration, we generated a unique transgenic mice model with constitutive overexpression of Rspo3 protein, utilizing the Cre-LoxP system under the skin–specific keratin-14 (K14) promoter (Figure 1A). For this purpose, we crossed three different mouse lines: K14Cre mice in which promoter activity commences in developing epithelia from approximately embryonic day 9 (E9) and continues to drive Cre expression in adult epidermis and hair follicles (Vasioukhin et al., 1999; Kobielak et al., 2003); CAG-lox-STOP-lox-Rspo3 mice for *Rspo3* transgene overexpression (Cambier et al., 2014); and ROSA26-lox-STOP-lox-eYFP (yellow fluorescent protein) mice (Srinivas et al., 2001), which allowed for the indication of the genetic recombination, and the permanent labeling of K14-derived tissue, as well as tracking of their progeny using YFP (Figure 1A). The expression pattern of YFP and K14 in Rspo3GOF mice was demonstrated by immunofluorescent staining of back skin, performed at P18 (Figure 1B).

Mice genotype was verified by PCR genotyping using primers specific for K14Cre, Rspo3, and YFP (Figure 1C). Moreover, the overexpression of Rspo3 in this model was confirmed by Western blot analysis of the whole skin lysates isolated from P7 Rspo3GOF and Con mice (Figure 1D).

To examine the physiological expression of *Rspo3*, mRNA in skin epidermis and hair follicles during early development, we used recently published publicly available single-cell RNA-seq (scRNA-seq) data (Qu et al., 2022). Our reanalysis of this scRNA-seq data identified a cluster representing a population of keratinocytes in the epidermis with *Rspo3* mRNA expression during the formation and growth of mouse hair follicle epithelial placode, at E13.5, E14.5, and hair germ stage at E15.5. However, the expression of *Rspo3* is successively decreasing during these three embryogenesis stages, and it was detected in 4.26% of cells at E13.5, 3.05% at E14.5, and diminished to 1.27% at E15.5 (Supplementary Figure S1A).

Further reanalysis of this cluster defined a subpopulation of cells with *Rspo3* mRNA expression localized in a subpopulation of cells positive for placode formation represented by *K14, Lef1,* and *Wnt10b* (Supplementary Figure S1B). Altogether, this analysis confirms that *Rspo3* is expressed in the wild-type epidermal cells from the embryonic stage when the hair placodes are formed.

### Rspo3 overexpression (Rspo3GOF) affects proper hair follicle development, resulting in very sparse hair coats in mice

To investigate the consequences of constitutive overexpression of Rspo3 in the epidermis and its appendages during HF development, we initially focused on general observation of the skin and hair phenotype of the transgenic Rspo3GOF mice compared to Con during different HF developmental stages from P2 to P43 (Figure 1E). Rspo3GOF mice exhibited a distinct and persistent phenotype characterized by a very sparse hair coat observed from early postnatal morphogenesis up to the end of their life. These observations suggested that the Rspo3 overexpression disturbed hair morphogenesis and affected hair growth. Additionally, we noted a mild exfoliation of the epidermis, suggesting alternations in the epidermal differentiation (Figure 1E). To assess, whether proper epidermal differentiation occurs in the Rspo3GOF mice, we conducted immunofluorescence staining of the back skin of Rspo3GOF and Con mice, targeting markers for epidermal layers at P14 (Supplementary Figure S2), as the most significant exfoliation was noted at that time point (Figure 1E, P14). However, we did not detect any changes in the expression of markers for the granular layer-Loricrin (Supplementary Figure S2A), basal layer-K14 (Supplementary Figure S2B), and a suprabasal layer-Keratin 10 (Supplementary Figure S2C). In order to investigate if overexpression of Rspo3 influences the formation of the sebaceous glands, we performed Oil Red staining of lipids in back skin sections of Con and Rspo3GOF mice at P18. There was no detectable differences between the sebaceous glands in Rspo3GOF mice and Con (Supplementary Figure S2D).

### Disturbed cycle with decreased length and amount of hair follicles as a result of Rspo3 overexpression

**FIGURE 2:**
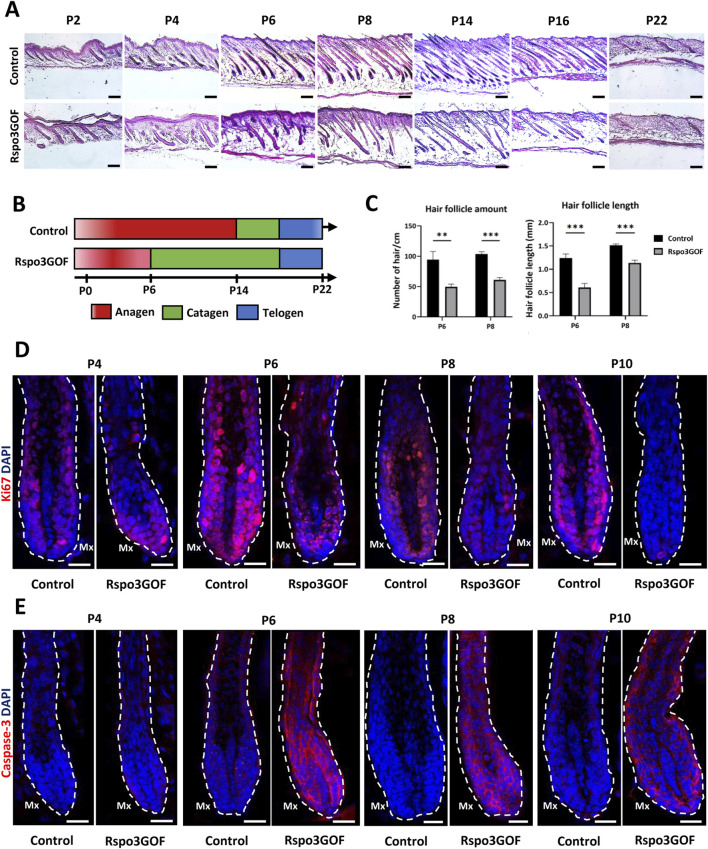
Rspo3GOF mice show changes in the hair follicle cycle. **(A)** Hematoxyline & Eosine staining of Control and Rspo3GOF back skin, performed during the first hair cycle (P2-P22). Scale bar = 1,000 µm. **(B)** Scheme depicting perturbed hair follicle cycle in Rspo3GOF mice compared to Control. **(C)** Comparison of hair follicle amount and length between control and Rspo3GOF mice. The stars are intended to flag levels of significance: (**) *p*-value < 0.01, (***) *p*-value <0.001. Error bars ±SEM. Data are representative of at least three independent experiments. **(D, E)** Immunofluorescence staining of Control and Rspo3GOF back skin, targeting the cell proliferation marker - Ki67 **(D)** and the cell apoptosis marker - Caspase-3 **(E)**, performed during the first hair cycle (P4, P6, P8, P10). DAPI counterstaining was used to label cell nuclei. Scale bars, 20 µm.

To further explain the observed phenotype in Rspo3GOF mice, we performed Hematoxylin and Eosin staining (H&E) of back skin from Rspo3GOF and Con mice during the first postnatal (P) hair cycle from P2 to P22 (Figure 2A). H&E staining revealed some disturbances in Rspo3GOF mice compared to Con, including changes in HF morphology comprising their decreased amount and length already visible from P2 till P22 (Figure 2A). Detailed analysis confirmed the significance of these changes, with an overall HF length reduction by approximately 50% at P6 and 25% at P8, along with a decrease in HF amount by over 50% at both P6 and P8 in Rspo3GOF mice (Figure 2C). H&E staining also showed that by P8, Rspo3GOF HFs down-growth toward the underlying dermis and adipose tissue was less progressed with noticeable changes in the hair bulb morphology, which appeared smaller and underdeveloped with a few misoriented bulbs (Figure 2A). Additionally, we observed, starting at P8 and continuing to P14, an increasing number of HFs with characteristic narrower bulbs with structures resembling epithelial strands, suggesting catagen-like stages in Rspo3GOF HFs and overall abnormal hair cycle progression compared to controls (Figure 2A). Thus, to precisely distinguish hair cycle phases, immunostaining of back skin was performed, targeting the proliferation marker Ki67 (Figure 2D) and apoptotic marker Caspase 3 (Cas3) (Figure 2E). While Con HFs were proliferating in the anagen phase until P10, in Rspo3GOF HFs, we noticed that Ki67 staining of hair bulb matrix cells has been strongly decreased already at P6, becoming barely visible by P8 and completely absent by P10, suggesting precocious withdrawal from anagen phase (Figure 2D). Interestingly, when the strong reduction in proliferation by Ki67 staining in the hair bulb matrix was found at P6, at the same time point within the bulb structure, we already detected staining for Cas3 (Figure 2E). These observations, confirmed the rapid increase in Cas3 expression from P6 through P10 which inversely correlated with a substantial decrease in the number of proliferating cells in Rspo3GOF hair bulbs by Ki67 as compared to Con (Figures 2D, E). At P22 both Con and Rspo3GOF HFs were in the telogen phase, which is consistent with the normal HFs cycle course (Figure 2A). Based on these results, we created a schematic diagram illustrating that while Con mice displayed the normal hair cycle progression, overexpression of Rspo3 affects the proper length of the anagen phase resulting in its shortening along with precocious entry into catagen, leading to HF degeneration (Figure 2B).

### Rspo3 plays a crucial role in hair follicle morphogenesis and its over expression leads to impaired HF formation

**FIGURE 3:**
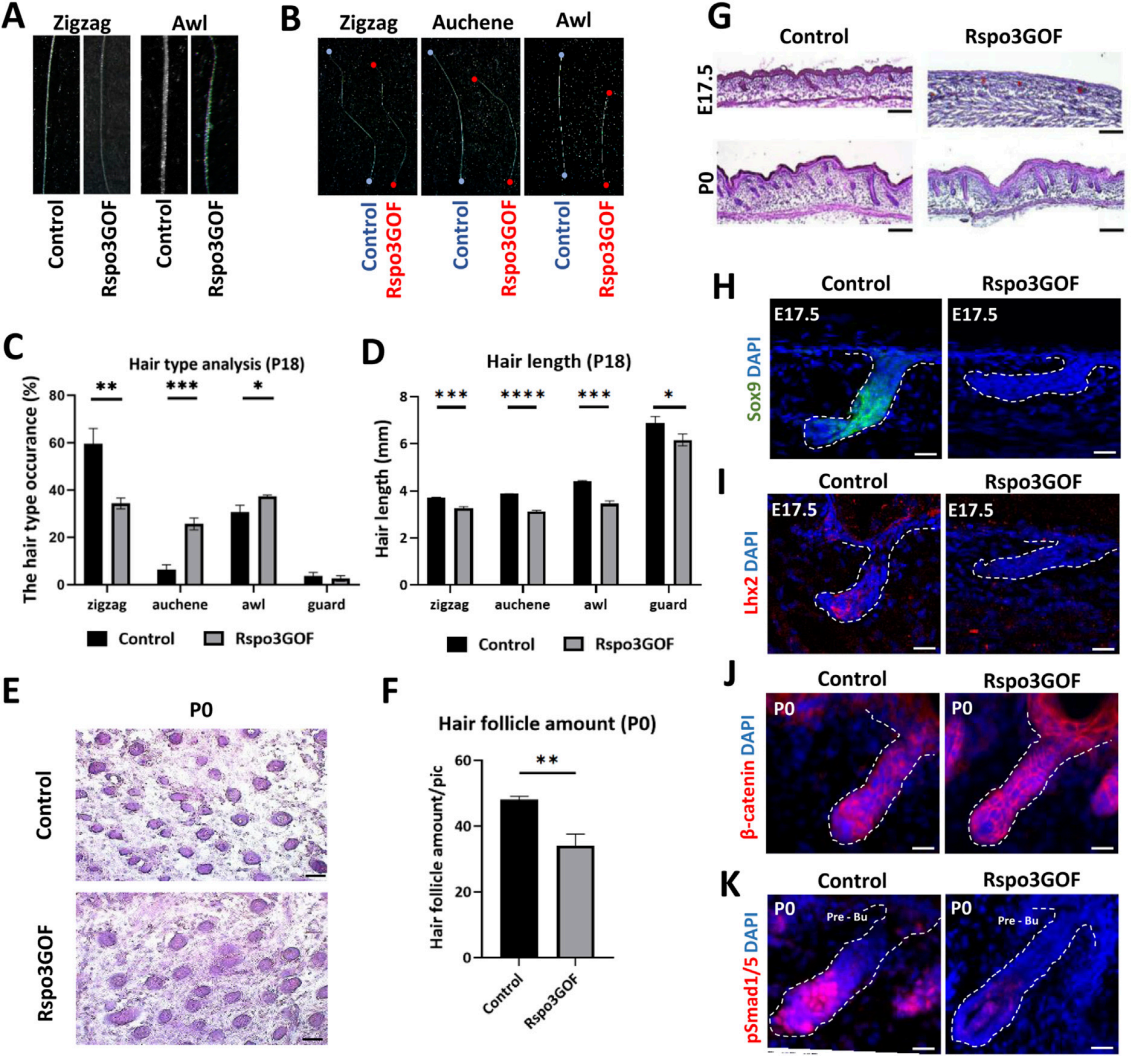
Rspo3GOF mice show changes in hair follicles formation, hair type occurrence and length. **(A)** Comparison of zigzag and awl hair shaft structure of Rspo3GOF and Control. **(B)** Comparison of length of different hair types from Control and Rspo3GOF mice. **(C, D)** Analysis of different hair types occurrence **(C)** and length **(D)** in Control and Rspo3GOF mice, conducted at P18. The values are mean ± standard deviation of a three replicates. Student’s t-test was used to test the significance: *p < 0.05; **p < 0.01; ***p < 0.001; ****p < 0.0001. Error bars ±SEM. Data are representative of at least three independent experiments. **(E)** Hematoxyline & Eosine (HE) staining of horizontally cut back skin from Control and Rspo3GOF mice at P0. Scale bar, 1,000 µm. **(F)** Analysis of hair follicle amount at P0 confirmed decreased hair follicle amount upon Rspo3 overexpression. Error bars ±SEM. Data are representative of at least three independent experiments. **(G)** HE back skin staining performed during the hair morphogenesis (E17.5; P0) show decreased hair amount and length in Rspo3GOF mice. Scale bar, 1,000 µm. **(H, I)** Immunofluorescence staining of Rspo3GOF and Control back skin at E17.5 targeting HFSCs markers Sox9 **(H)** and Lhx2 **(I)**. DAPI counterstaining was used to label cell nuclei. Scale bars, 20 µm. **(J, K)** Immunofluorescence staining of Rspo3GOF and Control back skin at P0 targeting β-catenin **(J)** and pSmad1/5 **(K)**. DAPI counterstaining was used to label cell nuclei. Scale bars, 20 µm.

Linking the previous studies demonstrating the role of Rspos in the activation of canonical Wnt pathway (Kazanskaya et al., 2004; Kim et al., 2005; Carmon et al., 2011; Carmon et al., 2012; de Lau et al., 2011; de Lau et al., 2014; Ruffner et al., 2012) to observed changes in hair follicle cycle, HFs amount, and length under Rspo3 overexpression (Figure 2), we decided to examine the hair morphogenesis in Rspo3GOF mice. The mouse hair coat comprises four types of hair: zigzag, guard, auchene, and awl, which are formed during three signaling waves (Duverger and Morasso, 2009; Chi et al., 2015). The first wave (E14.5) leads to guard hair formation, the second (E16.5) shows the plasticity in the created hair type and can result in awl or auchene hair, whereas zig zag hair are formed after the third signaling wave (E17.5-P0) (Schneider et al., 2009). Analysis of hair at the end of the first postnatal telogen (P18) revealed that all four types of hair were present in the dorsal skin of both Con and Rspo3GOF mice. However, the composition of the hair coat in Rspo3GOF mice differed from that of Con mice. We observed a significant decrease of over 40% in the occurrence of zigzag hair type, accompanied by an increased occurrence of both auchene hair by approximately 400%, and awl hair by around 20%, with no noticeable changes in guard hair (Figure 3C). In addition to changes in the proportions of hair types, Rspo3GOF mice also displayed an overall reduction in hair length, with zigzag hair decreasing by 12%, auchene by 19%, awl by 21%, and guard by 11% (Figures 3B, D). Moreover, we observed changes in the zigzag and awl hair shaft structure in Rspo3GOF mice compared to Con, including a reduction in the thickness and pigmentation (Figure 3A).

### Rspo3 overexpression (Rspo3GOF) affects proper pre-bulge formation for early HFSCs specification

Given the observed alternations in hair type occurrence resulting from Rspo3 overexpression, we hypothesized that Rspo3 may play a crucial role in regulating HFs morphogenesis. To investigate this, we conducted H&E staining at E17.5 and P0, revealing perturbed hair follicle formation under Rspo3 overexpression. While at E17.5 hair placodes were visible in Rspo3GOF, they down growth toward the dermis was much less progressed compared to Con (Figure 3G). At P0, although the HFs down growth appeared similar in Rspo3GOF and Con mice, Rspo3GOF exhibited polarity issues and a reduced number of follicles (Figure 3G). Additionally, we horizontally sectioned the back skin of P0 Con and Rspo3GOF mice (Figure 3E) and analyzed the HFs amount, revealing a decrease of approximately 30% in Rspo3GOF mice (Figure 3F). Further, we decided to examine whether Rspo3 influences the formation of HFSC population during the embryonic developmental stage, and we did not observe any expression of neither Sox9 (Figure 3H) nor Lhx2 (Figure 3I) in Rspo3GOF at E17.5. Then, by performing immunofluorescent staining targeting β-catenin (Figure 3J), we were able to show that an abnormal course of hair morphogenesis in Rspo3GOF mice results from the elevated expression of β-catenin along the entire hair structure when compared to controls, in which β-catenin was only present in the matrix cells of the hair bulb. Together with the elevated expression of β-catenin in Rspo3GOF mice, we demonstated that the staining against phosphorylated Smad1/5 (pSmad1/5) was reduced in this trangenic mice model compared to Con (Figure 3K). Taken together, our data shows that Rspo3 plays an important role in hair follicle morphogenesis and changes in its expression lead to impaired formation of HFs.

### Overexpression of Rspo3 influences the maturation of the hair follicle stem cell population in the bulge

**FIGURE 4:**
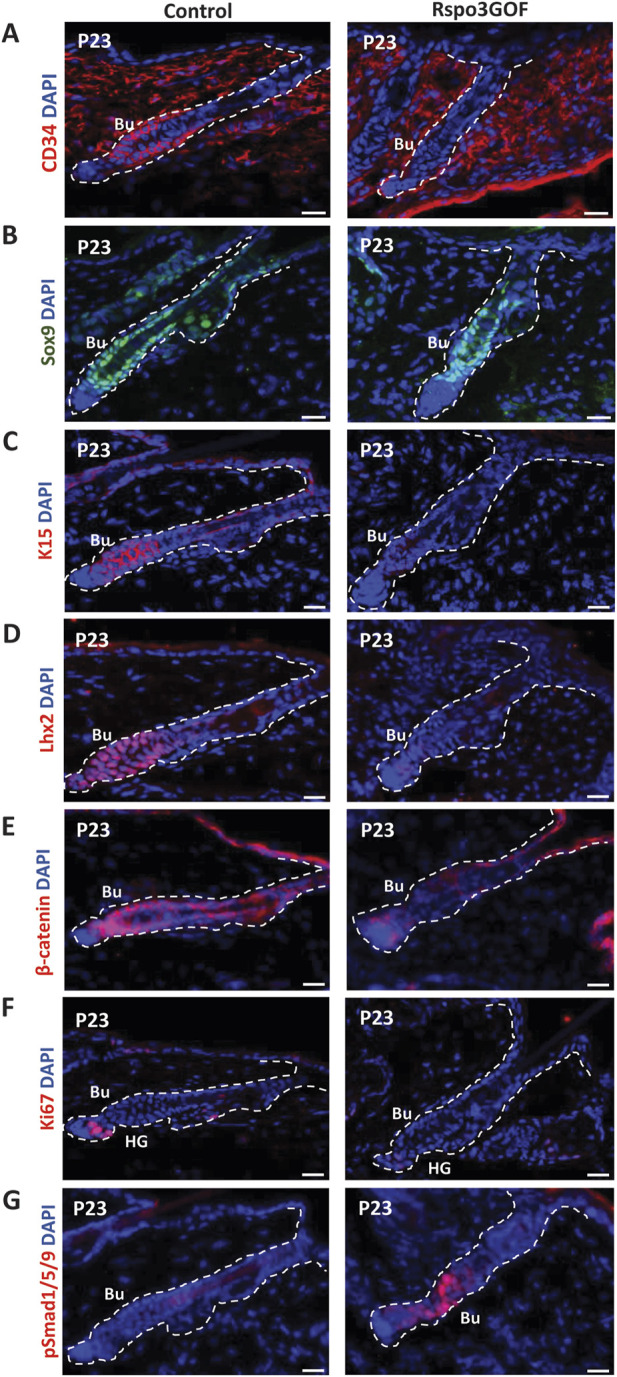
Rspo3GOF mice displayed immature bulge with a reduction of expression of specific hair follicle stem cells (HFSCs) markers and delayed telogen phase. Immunofluorescence staining of Rspo3GOF and Control back skin at P23 targeting hair follicle stem cells (HFSCs) markers: CD34 **(A)**, Sox9 **(B)**, K15 **(C)**, Lhx2 **(D)**, as well as, β-catenin **(E)**, and proliferation marker, Ki67 **(F)**, and pSmad1/5/9 **(G)**. DAPI counterstaining was used to label cell nuclei. Scale bars, 20 µm. Abbreviation: Bu, bulge; HG, hair germ.

To study the status of the HFSC population in the bulge during postnatal development, we performed immunofluorescence staining of back skin at P23, during the first postnatal telogen-to-anagen transition, using various HFSC markers (Figure 4). Whereas Con mice exhibited a prominent bulge area marked by CD34, a well-known indicator of HFSCs, seen at first postnatal telogen in quiescent HFSCs at P18, surprisingly we did not detect any expression of this marker in the bulge region of Rspo3GOF mice (Figure 4A). However, in contrast, we observed that the level of another HFSCs marker, Sox9 was expressed at the same level in both, Con and Rspo3GOF (Figure 4B). To further assess the status of the HFSC population we decided to use another well-characterized marker for bulge HFSCs, keratin 15 (Liu et al., 2003; Morris et al., 2004). While Con mice had K15-labelled HFSCs in the bulge area, we did not detect K15 expression in HFSCs of Rspo3GOF (Figure 4C). Furthermore, Rspo3GOF mice exhibited also a significant reduction of another, well-known HFSCs marker in the bulge - Lhx2, when compared to a well-established signal in Con (Figure 4D). Interestingly, although we observed a high level of β-catenin staining in the bulge and hair germ during early telogen-to-anagen transition at the onset of the hair regeneration cycle at P23 in control, in contrast, there was just a faint visible signal from β-catenin staining in Rspo3GOF skin sample (Figure 4E). As we previously described, constitutive overexpression of Rspo3 in the skin and its appendages resulted in disturbed progression of the first HF cycle (Figure 2). Therefore, we performed immunofluorescent staining with proliferation marker-Ki67, to assess whether the transition from the first telogen to the second anagen occurs normally in these mice. The substantial reduction in Ki67 signal that we observed (Figure 4F), led us to conclude that Rspo3GOF mice experienced a delayed onset of the second HFs cycle due to the HFSC population not reaching full maturity. Moreover, we demonstrated that at that time point, Rspo3GOF mice displayed elevated expression of phosphorylated Smad1/5/9 (pSmad1/5/9) in the bulge compared to Con (Figure 4G).

### Prolonged immaturity of bulge hair follicle stem cell population in old Rspo3GOF mice

**FIGURE 5:**
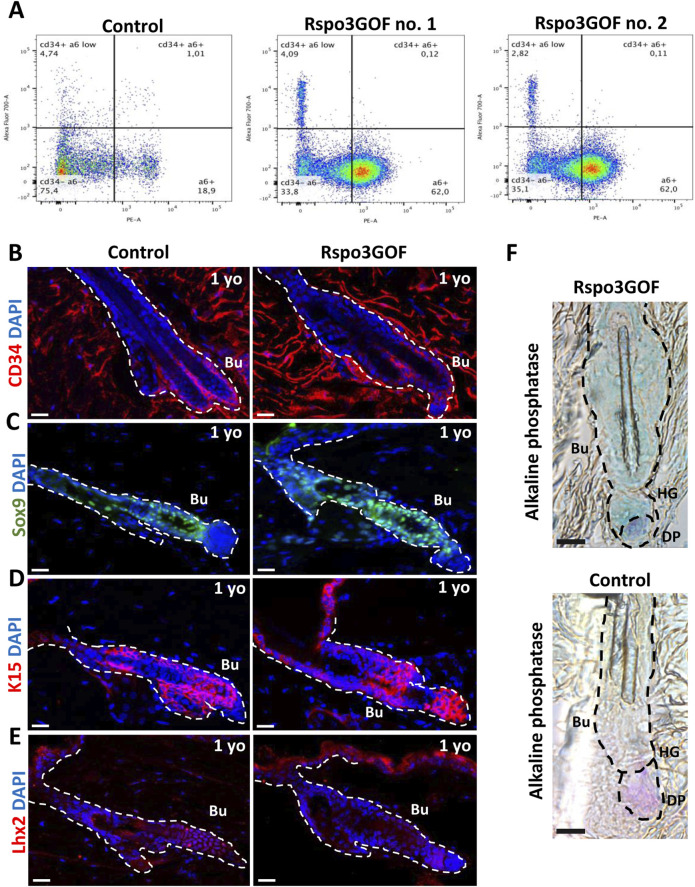
Long-term Rspo3 overexpression sustained prolonged immaturity of bulge HFSCs in aged mice. **(A)** FACS sorting results depicting population of keratinocytes isolated from the whole epidermis of one-year old (1 year) Control and Rspo3GOF mice. The CD34^+^, α6-integrin positive cells represented 1% of the whole keratinocytes population in Control and 0.1% in Rspo3GOF mice. **(B–D)** Immunofluorescence staining of 1 yo Control and Rspo3GOF mice back skin, targeting HFSCs markers: CD34 **(B)**, Sox9 **(C)**, K15 **(D)**, Lhx2 **(E).** DAPI counterstaining was used to label cell nuclei. Scale bars, 20 µm. **(F)** Alkaline phosphatase staining (purple) identified a presence of proper dermal papillae (DP) localization in 1 yo Rspo3GOF mice. Scale bar, 500 µm. Abbreviation: Bu, bulge; HG, hair germ.

These results prompted us to investigate whether the observed delay in the development of HFSCs maturation in the bulge that we detected is permanent and persists throughout the lifespan of Rspo3GOF mice. To address this, at first, we examined the global population of HFSCs by performing Fluorescence-activated Cell Sorting (FACS), sorting CD34 and α6-integrin positive cells from the whole epidermis of one-year-old Con and Rspo3GOF mice (Figure 5A). The obtained results revealed that these cells accounted for 1% of the total population in Con mice, while in Rspo3GOF mice, CD34 and α6-integrin positive cells represented only 0.1% (Figure 5A). To validate this observation and to further study the HFSC population, we performed immunofluorescence staining of the back skin of one-year-old Con and Rspo3GOF mice (Figures 5B–E). The results indeed revealed diminished expression of CD34 in Rspo3GOF mice (Figure 5B). Additionally, Rspo3GOF mice exhibited a similar level of Sox9 expression compared to Con mice (Figure 5C), consistent with our observation during the first postnatal telogen at P23 (Figure 4B). Interestingly, although we previously observed a complete lack of K15 marker expression in the bulge region at P23 (Figure 4C), we noted expression of the K15 marker in the bulge region of one-year-old Rspo3GOF mice similar to that observed in Con mice (Figure 5D). Furthermore, Lhx2 expression remained significantly reduced in one-year-old Rspo3GOF mice (Figure 5E), comparable to previous observations at P23 in young Rspo3GOF mice compared to Con (Figure 4D). Additionally, by performing alkaline phosphatase staining, we confirmed the presence of proper localization and morphology of dermal papillae (DP), which remained attached to hair germ (HG) and bulge region in one-year-old Rspo3GOF mice alike in Con mice (Figure 5F). We did not also notice any alternations in the hair cycle of Rspo3GOF mice at this time point, as the immunofluorescence staining targeting Ki67 (Supplementary Figure S3A) and Caspase-3 (Supplementary Figure S3B) showed a lack of expression of these markers in both Con and Rspo3, indicating that the hair follicles of these mice were in the telogen stage. In summary, our finding showed that the effect of Rspo3 overexpression on HFSCs is long-term and this population still demonstrates prolonged immaturity of bulge region both in young and aged mice.

### Rspo3 overexpression impacts keratinocyte proliferation and motility *in vitro*

**FIGURE 6:**
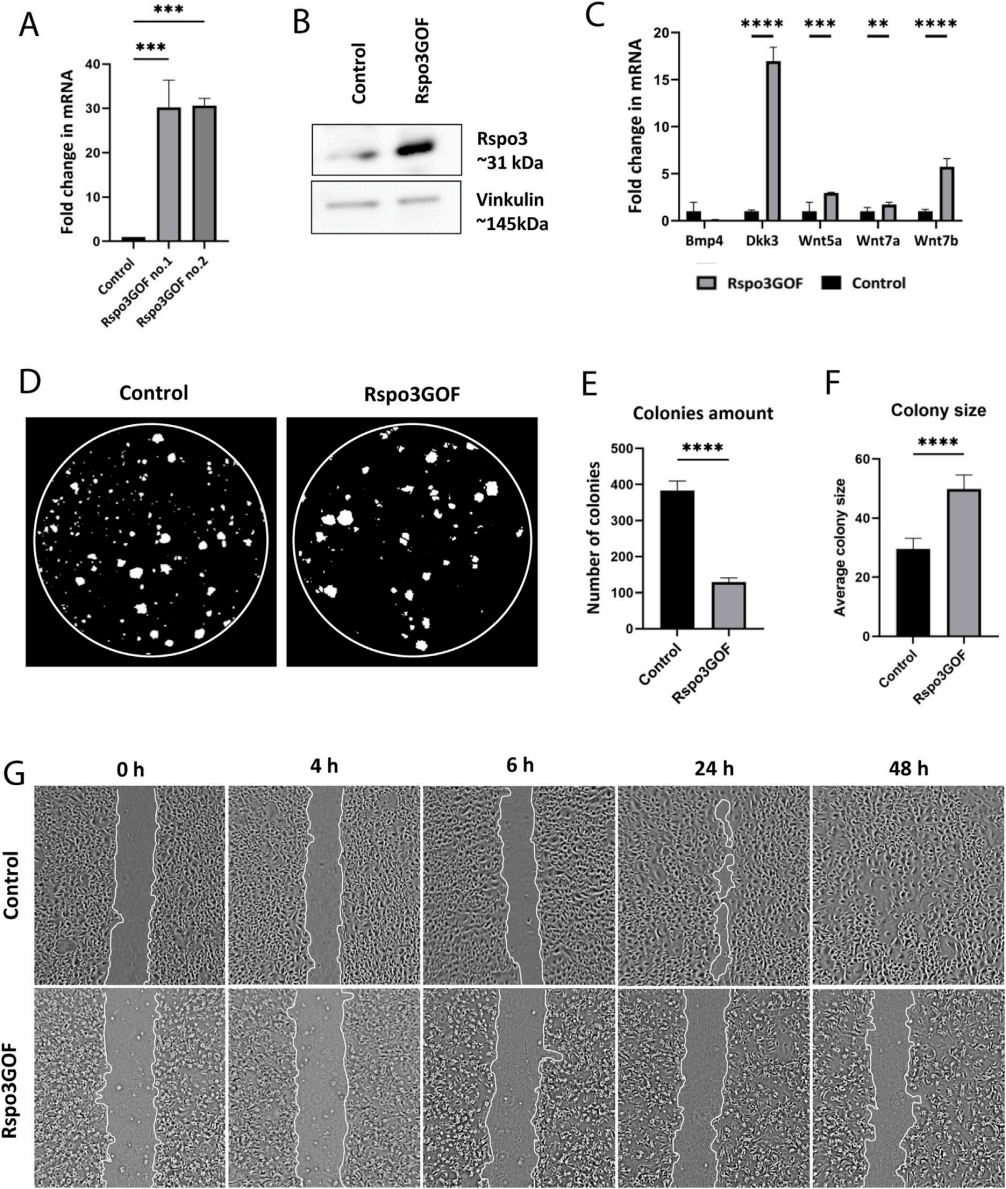
Keratinocytes with Rspo3 overexpression exhibit decreased ability to proliferate and migrate. **(A, B)** Rspo3 overexpression in keratinocytes was confirmed by RT-qPCR analysis. Error bars ±SEM. Data are representative of at least three independent experiments **(A)** and Western blot **(B)**. **(C)** RT-qPCR analysis depicting increased expression of Dkk3, Wnt5a, Wnt7a, Wnt7b, and decreased expression of Bmp4 in Rspo3GOF keratinocytes compared to Control. Results were normalized to β-actin. Error bars ±SEM. Data are representative of at least three independent experiments. **(D)** Representative pictures from Colony Formation Assay (CFA), performed on control and Rspo3GOF keratinocytes and taken after 9 days of culture. **(E)** CFA analysis revealed decreased ability of Rspo3GOF keratinocytes to formation of colonies. **(F)** CFA analysis showed increased size of colonies formed by Rspo3GOF keratinocytes compared to Control. Error bars ±SEM. Data are representative of at least three independent experiments. **(G)** Representative pictures of Control and Rspo3GOF keratinocytes from the Wound Healing assay, taken during different time points from the start (0, 4, 6, 24, 48 h).

Next, we isolated keratinocytes from P7 Rspo3GOF and Con mice and examined their ability to proliferate and participate in skin physiology by performing *in vitro* assays. Both Con and Rspo3GOF keratinocyte lines were able to undergo multiple passages (>10 passages). The overexpression of Rspo3 in cultured keratinocytes was confirmed in two separate cell lines by RT-qPCR (Figure 6A) and Western blot analysis (Figure 6B). *Rspo3* mRNA expression was approximately 30 times higher in both Rspo3GOF cell lines compared to Con (Figure 6A). Furthermore, by performing RT-qPCR, we demonstrated that the *Rspo3* overexpression acts through the canonical Wnt pathway in keratinocytes. Compared to control, Rspo3GOF keratinocytes had increased expression of *Wnt5a, Wnt7a, Wnt7b, and Dkk3*, together with decreased expression of *Bmp4* (Figure 6C).

Then, we performed the Colony Formation Assay (CFA) which revealed changes in the capability of Rspo3GOF keratinocytes to form colonies. We observed a reduced number of colonies created after 9 days by Rspo3GOF keratinocytes compared to Con (Figure 6D) with a more detailed analysis showing an amount reduction of over 65% (Figure 6E). Interestingly, the average colony size created by Rspo3GOF keratinocytes was approximately 40% larger than that of Con (Figure 6F). To verify whether changes in colony size result from increased keratinocytes differentiation, we cultured both Con and Rspo3GOF keratinocytes in increasing calcium concentrations (0.05 mM Ca^2+^, 0.3 mM Ca^2+^, 0.8 mM Ca^2+^) and therefore performed immunofluorescent staining targeting the keratinocyte differentiation markers (Supplementary Figure S4). However, we did not notice changes in the expression of any of the markers: Keratin10 (Supplementary Figure S4A), Keratin14 (Supplementary Figure S4B), and Loricrin (Supplementary Figure S4C). Additionally, we revealed that the observed bigger average colony size formed by Rspo3GOF keratinocytes was also not caused by an increased number of cells in individual colonies, as precise analysis pointed to a decreased number of cells per colony in particular days of CFA (Supplementary Figure S5B). Therefore, we concluded that these changes might simply result from a difference in the morphology of Rspo3GOF keratinocytes compared to Con, since as already after 3 days of the test, Rspo3GOF keratinocytes were larger compared to Con (Supplementary Figure S5A). Overall, CFA results revealed decreased proliferation of Rspo3GOF keratinocytes compared to Con, and these data were also supported by results of immunofluorescent staining against proliferation marker, Ki67 (Supplementary Figure S5C), with the analysis showing a reduction in the proliferating cell number by almost 30% (Supplementary Figure S5D).

Furthermore, to study the motility of Rspo3 and Con keratinocytes, we conducted a Wound-Healing assay, using mitomycin C - blocked keratinocytes. Whereas Con cells were able to fully cover the gap in 48 h from seeding, Rspo3GOF keratinocytes failed to cover the wound at all (Figure 6G). Detailed analysis confirmed a decrease in the percentage of wound closure (Supplementary Figure S5E). Taken together, obtained results suggest impaired proliferation and migration of keratinocytes, as well as abnormal cell morphology, under the constitutive Rspo3 overexpression.

## Discussion

### Physiological Rspo3 expression correlates with canonical Wnt signaling during early morphogenesis

Our re-analysis of available single-cell RNA-seq data (scRNA-seq) revealed the dynamic of the physiological expression pattern of *Rspo3* during early embryonic development, particularly in epidermal keratinocytes. We observed a peak in expression during hair follicle placode formation at E13.5 and 14.5, followed by a decline at E15.5 during hair germ formation, emphasizing its active physiological role in hair morphogenesis (Supplementary Figure S1). This finding is significant in the context of the previously proposed role of Rspo3 in enhancing Wnt signaling (Carmon et al., 2011; Carmon et al., 2012; de Lau et al., 2014; Ruffner et al., 2012; de Lau et al., 2011).

Our findings align with previous studies that established canonical Wnt signaling is a key player in HF development, as it triggers the formation of placodes, which in turn give rise to the hair germ (Hardy, 1992; Millar, 2002; Andl et al., 2002; Saxena et al., 2019; Rishikaysh et al., 2014). Consistent with this, prior studies have shown that mice lacking the gene encoding β-catenin for the canonical Wnt pathway failed to form hair placodes (Huelsken et al., 2001). Conversely, overexpression of β-catenin in the skin of transgenic mice led to *de novo* formation of hair follicles with the proper structure, including sebaceous glands and dermal papillae (DP) (Gat et al., 1998). The expression pattern of canonical Wnt signaling, visualized in TOPGAL reporter mice, also supports our observations, with increased Rspo3 expression during placode formation and a subsequent decrease during hair germ formation (DasGupta and Fuchs, 1999).

### The Rspo3 overexpression promotes induction of canonical Wnt-dependent hair type: auchene and awl during morphogenesis, although the overall hair amount remains reduced

Rspo3 overexpression during hair morphogenesis resulted in significant changes in the formation of specific hair types (Figure 3C), despite an overall reduction in hair amount observed in Rspo3GOF mice (Figure 3F). We observed promotion of auchene and awl hair types, with increased development by over 400% and 20%, respectively. Previous research has demonstrated that awl and auchene hair are induced during the second wave of hair morphogenesis at E16.5 and are characterized by specific dermal papilla cells positive for Sox2-GFP and CD133. These cells predominantly express genes involved in Wnt, BMP, and FGF signaling (Driskell et al., 2009). Additionally, during the formation of awl and auchene hair types, the placodes were marked by TOPGAL and Lef1 expression, confirming the crucial role of the canonical Wnt pathway in the second wave of hair morphogenesis (DasGupta and Fuchs, 1999). In contrast, zigzag hair, formed during the third wave of morphogenesis are characterized by Sox2-GFP negative and CD133 positive dermal papilla cells and exhibit a high representation of Shh, IGF, Notch, and integrin signaling pathways (Driskell et al., 2009).

Thus, our results indicate that Rspo3 overexpression enhanced canonical Wnt signaling during early morphogenesis and promoted the generation of Wnt-dependent auchene and awl hair types. Conversely, zigzag hair, formed during the third wave of hair regeneration, were reduced by over 40%, suggesting a Wnt-independent mechanism for their formation (Figure 3C). Since zigzag hair constitute approximately 60% of all hair follicle types, therefore the overall hair amount remained reduced in Rspo3GOF mice. These findings suggest that Rspo3, in conjunction with enhancing Wnt signaling, may play a pivotal role in orchestrating the initiation of distinct hair types during hair morphogenesis.

### Rspo3 overexpression enhances β-catenin expression in the hair matrix, resulting in a premature anagen-to-catagen transition with a shortened growth phase and decreased overall length of all hair types

Here, we demonstrated that mice with constitutive overexpression of *Rspo3* gene in the skin and its appendages exhibited a stable, distinct phenotype characterized by sparse hair and visible bald areas from the early stages of development (Figure 1E). Furthermore, the examination of the hair cycle progression in Rspo3GOF mice revealed a shorter anagen growth phase and premature catagen onset, which began already at P6 (Figures 2A, B, D, E), leading to a reduction in overall hair length (Figure 2C). Additionally, our results indicated that hair matrix cell proliferation, as indicated by Ki67 staining, prematurely declined at P6 and was almost completely terminated around P8 (Figure 2D). Rspos are the potent enhancers of the canonical Wnt pathway mediated by Lef1/β-catenin complexes in hair matrix progenitors responsible for switching these cells from the proliferation to differentiation into precortical and subsequently cortical layers of the hair shaft. Indeed, immunostaining revealed an increased level of β-catenin throughout the entire hair follicle, including the matrix and epidermis in Rspo3GOF as compared to the control (Figure 3J), which coincided with the pattern of Rspo3 overexpression under K14-Cre reporter in the skin (Figure 1B). Consequently, the earlier termination of proliferation in the hair matrix seen in Rspo3GOF mice at P6-P8 (Figure 2D) suggests that the observed increased expression level of β-catenin corresponds with precocious activation of canonical Wnt signaling. This, in turn, leads to faster differentiation of matrix progenitors into precortical cells, which subsequently create the cortex, resulting in the formation of shorter hair shafts along with medulla and cuticle layers. Furthermore, our *in vitro* results supported these observations since impaired proliferation and migration of keratinocytes (Figures 6D, E, G) might reminiscence changes observed in hair matrix progenitors *in vivo* (Figure 2D).

### Rspo3 overexpression enhanced β-catenin expression, impairing the formation and maturation of hair follicle stem cell population during hair morphogenesis

Our study of the HFSC population revealed a significant effect of Rspo3 overexpression on pre-bulge formation and maturation during hair morphogenesis. Initially, we observed a delay in the appearance of expression of the well-characterized pre-bulge markers Sox9 and Lhx2 for early HFSCs formation in Rspo3GOF mice at E17.5 during hair germ stage (Figures 3H, I) (Nowak et al., 2008; Kandyba et al., 2014; Vidal et al., 2005; Rhee et al., 2006). The delay in the formation of pre-bulge with the HFSC population (Figures 3H, I), can be attributed to the prolonged and expanded non-physiological activation of the canonical Wnt signaling. This is evidenced by the overlapping expression of Rspo3 (Figure 1B) and enhanced β-catenin in the entire hair follicle and epidermis (Figure 3J), extending beyond the physiological restriction of the hair placode, confirmed earlier in TOPGAL reporter mice (DasGupta and Fuchs, 1999). Interestingly, we found that increased β-catenin staining in Rspo3GOF during the hair follicle formation correlated with downregulation of pSmad1/5 in pre-bulge compare to control (Figures 3J, K), suggesting delayed in establishment of the quiescent HFSCs. That observation is consistent with previously published observation in double knockout for pSmad1/5, where the formation of pre-bulge has been missing (Kandyba et al., 2014). These findings also align with previously published data indicating that displaced suprabasal daughter cells in the hair placode, expressing a low level of Wnt, become future Sox9 positive HFSCs sensitive to paracrine Shh signals. Conversely, basal daughter cells in the hair placode express a high level of Wnt and are a source of Shh, responsible for the slow-cycling maintenance of stem cells (Ouspenskaia et al., 2016). Additionally, Lhx2-KO mice showed delayed growth of primary hair placodes and reduced numbers of HFs (Rhee et al., 2006; Tomann et al., 2016), which is consistent with observations in our Rspo3GOF model at this early time point (Figures 3E–G). Furthermore, hair germs of Rspo3GOF mice lacked expression of Sox9 (Figure 3H), which is essential for maintaining the growth of postnatal follicles during the hair cycle as Sox9 positive cells give rise to developing hair follicles and are crucial for maintaining HFSCs (Nguyen et al., 2018).

Subsequently, we investigated how prolonged overexpression of Rspo3 affects HFSCs beyond the hair morphogenesis stage. Physiologically, after hair morphogenesis is completed, a mature bulge should have been established during the first telogen phase at P18. However, in Rspo3GOF mice, we observed that only the level of Sox9 expression was restored at the first telogen-to-anagen transition at P23 (Figure 4B). Interestingly, the expression of the rest of the HFSC markers, including CD34, K15, and Lhx2, was still impaired (Figures 4A, C, D), indicating that Rspo3GOF mice exhibited a delay in the pre-bulge formation during morphogenesis and sustained prolonged immaturity of the HFSC population in bulge region postnatally.

Thus, our observations provided new insight into the role of Rspo3 on the formation of pre-bulge and bulge during hair morphogenesis, as previous studies predominantly focused on investigating the effect of Rspos, on telogen-to-anagen transition with the induction of precocious and extended anagen phase (Hagner et al., 2020; Chen et al., 2023; Smith et al., 2016). However, it is essential to note that these previous studies primarily involved the subcutaneous injection of Rspos during the telogen phase when the hair morphogenesis was fully completed and a mature bulge with HFSCs was already established. In contrast, the overexpression of Rspo3 in our mice model addressed the question of its role starting from the very early stages of hair morphogenesis.

### Immature hair follicle stem cell population in Rspo3GOF mice decreased β-catenin expression level, impairing telogen-to-anagen hair cycle progression postnatally

Contrary to our observation of an increased β-catenin expression in Rspo3GOF mice during hair morphogenesis (Figure 3J), we surprisingly discovered that after a bulge with immature HFSCs is established postnatally, β-catenin expression is downregulated during the telogen-to-anagen transition at P23 (Figure 4E). This suggests the downregulation of the canonical Wnt pathway at this hair cycle stage. Indeed, by performing immunofluorescent staining targeting proliferation marker, Ki67, we demonstrated that during the postnatal development, Rspo3GOF mice exhibit a diminished hair cycle progression with impaired telogen-to-anagen transition and reduced HFSCs proliferation (Figure 4F). Moreover, we observed that downregulation of β-catenin expression correlated with upregulation of pSmad1/5/9 in Rspo3GOF mice at P23, explaining the functional delay in the onset of anagen and linking it directly to the previously well-described role of canonical BMP signaling in maintaining quiescence of HFSCs in bulge (Figures 4E–G) (Kobielak et al., 2007; Kandyba et al., 2013; Kandyba et al., 2014).

Given that Rspo3GOF mice displayed a phenotype characterized by very sparse hair coat and visible bald areas throughout the lifespan (Figure 1E), our studies also confirmed delayed maturation of the HFSC population in aged mice by still diminished expression of HFSC markers in one-year-old Rspo3GOF. We were able to reveal that the HFSC population sustained incomplete maturation in aged Rspo3GOF mice by reduction of CD34 (Figures 5A, B) and Lhx2 (Figure 5E) markers in the bulge area, along with nearly normal expression of Sox9 and K15 (Figures 5C, D, respectively). These observed abnormalities in reaching full maturity by HFSCs of Rspo3GOF mice most likely contribute to the sustained phenotype of Rspo3GOF mice through their lifespan and impair their potential for proper activation. This impairment is consistent with previous results showing that Lhx2 is required for anagen induction (Törnqvist et al., 2010) and that HFSCs lacking CD34 expression had a delayed anagen onset (Trempus et al., 2007). This suggested that Rspo3 overexpression results in immaturity of HFSCs, leading to delayed entry to anagen (Figure 4F). These results differ significantly from those published before, which show that injection of recombinant forms of Rspos promotes the activation of HFSCs (Smith et al., 2016; Chen et al., 2023). One possible explanation is that impaired HFSCs maturation by overexpression of Rspo3 likely affects hair cycle dynamics, compromising hair follicle cycle progression due to decreased β-catenin expression levels (Figures 4E, F).

Interestingly, we observed different outcomes concerning Wnt signaling due to early overexpression of Rspo3 during morphogenesis, as we first observed increased β-catenin expression during pre-bulge formation, and then downregulation of the β-catenin expression in immature bulge. These discoveries suggest that Rspo3 overexpression influences HFSCs formation in pre-bulge during hair morphogenesis via activation of the canonical Wnt pathway (Figures 3H–J) which as consequence might reduce canonical BMP signaling (Figure 3K). Moreover, this early canonical Wnt activation in pre-bulge had long-term repercussion in the first postnatal hair regeneration cycle, characterized by incomplete maturation of HFSCs in the adult bulge of Rspo3GOF mice (Figures 4A–D, 5A–E), with impaired β-catenin expression (Figure 4E), as results of pSmad1/5/8 with canonical BMP signaling activation (Figure 4G). These observations support the notion that Rspo3 is a crucial modulator of hair follicle morphogenesis and hair cycle, working through enhancing the canonical Wnt signaling by activating β-catenin (Figure 3J). Additionally, the results obtained from RT-qPCR analysis conducted on keratinocytes isolated from mice at P7, depicted an increased expression of *Wnt5a*, *Wnt7a*, and *Wnt7b*, further suggesting the enhancement of the activity of the canonical Wnt ligands in Rspo3GOF mice during the early development. Interestingly, Rspo3GOF keratinocytes exhibited a significant increase in expression of *Dkk3* (Lim et al., 2016). Both Dkk3 and these Wnt ligands were previously discovered to be a part of the intrinsic HFSCs oscillator which constantly operates in stem cells, introducing a proper balance between hair cycle activation and quiescence, as well as maintaining stem cell homeostasis (Kandyba et al., 2013; Kandyba and Kobielak, 2014; Daszczuk et al., 2020).

Collectively, our results suggest that early activation of the canonical Wnt pathway in Rspo3GOF during morphogenesis might induce a precocious feedback mechanism which accelerates a faster transition from anagen to catagen, establishing quiescent immature HFSCs in adult bulge during the first telogen. Thus, we speculate that this precocious quiescence state of immature HFSCs in Rspo3GOF might be maintained by BMP signaling and non-canonical Wnt signaling. Then paradoxically overexpression of Rspo3 during the quiescent state of immature HFSCs, might control the rotation of the Wnt-Frizzled receptor, enhancing the non-canonical Wnt telogen phase (Hao et al., 2012; Zebisch et al., 2013; Kandyba et al., 2013) Therefore, it will be interesting to address further and determine the Rspo3 role in regulating of the switch between the non-canonical and canonical pathway in the future.

Our findings shed light on crucial aspects of hair follicle biology and offer promising avenues for the development of therapeutic interventions targeting hair loss disorders. The regulation of HFSCs by Rspo3, along with its interaction with Wnt signaling pathways, presents potential targets for modulating hair growth and regeneration. Through our research, we have elucidated the dual role of Rspo3 in enhancing canonical Wnt signaling during early morphogenesis while also promoting quiescence in HFSCs. This deeper understanding allows us to better grasp the intricate regulatory mechanisms governing hair cycle dynamics. Moreover, the observed alterations in keratinocyte function and HFSC maturation in Rspo3GOF mice underscore the importance of precise regulation of Rspo3 expression for maintaining healthy hair follicles. This insight holds significant therapeutic implications, as it suggests that modulating Rspo3 levels or its downstream signaling pathways could potentially restore normal hair growth patterns.

Furthermore, existing research has explored therapeutic approaches involving other members of the R-spondin family. For instance, research from the last decade demonstrated that R-spondin1 and R-spondin2 can enhance hair regeneration, stimulate hair growth, and regulate hair follicle cycles by targeting the Wnt/β-catenin pathway and dermal progenitor functions (Li et al., 2016; Smith et al., 2016; Hagner et al., 2020; Hashimoto et al., 2022; Chen et al., 2023). These studies collectively underscore the potential of targeting R-spondins for hair growth stimulation and highlight the diverse strategies being explored in this area of research.

## Limitations

This study has several limitations. First, the transgenic mouse model with constitutive Rspo3 overexpression does not replicate natural dynamic expression patterns, affecting multiple cell types and complicating specific cell-type analysis. Additionally, focusing solely on Rspo3 overlooks interactions with other signaling pathways. Our *in vitro* conditions do not fully mimic the *in vivo* environment, and we did not extensively explore the functional outcomes related to changes in hair type proportions and HFSC maturation. Differences between murine and human hair biology also necessitate validation in human tissues. Lastly, potential off-target effects of Rspo3 overexpression were not fully characterized. Future research addressing these limitations is crucial for a comprehensive understanding of Rspo3’s role and therapeutic potential.

## Conclusion

In summary, our study underscores the critical role of Rspo3 in hair follicle development and hair cycle regulation. By comparing our results with previous studies, we highlight the unique effects of early Rspo3 overexpression on HFSC formation and hair cycle dynamics. These findings pave the way for future research on therapeutic interventions targeting Rspo3 and Wnt signaling to treat hair loss conditions. Our comprehensive analysis provides compelling evidence for Rspo3’s involvement in coordinating HF development, hair cycle progression, and HFSC regulation, offering new avenues for understanding and manipulating hair biology.

## Data Availability

The original contributions presented in the study are included in the article/Supplementary Material, further inquiries can be directed to the corresponding author.

## Supplementary material

Supplementary Figure S1 Expression of Rspo3 gene in the epidermal cells during hair follicle epithelial placode formation and growth. **(A)** Expression levels of Rspo3 gene among the epidermal cells in E13.5, E14.5, and E15.5. **(B)** Uniform Manifold Approximation and Projection (UMAP) representations of the epidermal population in E13.5, E14.5, and E15.5, showing expression of Rspo3 and following placode markers: Keratin 14 (Krt14), Lef1, and Wnt10b.

Supplementary Figure S2 Constitutive overexpression of Rspo3 in the skin and its appendages do not affect the epidermal differentiation process. Immunofluorescence staining of Rspo3GOF and Control back skin at P14 targeting the epidermal layers’ markers: Loricrin **(A)** for granular layer, Keratin14 **(B)** for basal layer, and Keratin10 **(C)** for suprabasal layer. DAPI counterstaining was used to label cell nuclei. Scale bars, 50 μm. **(D)** Oil red staining of lipids in back skin sections of Control and Rspo3GOF mice performed at P18. Scale bars, 200 μm. Abbreviation: SG, sebaceous gland.

Supplementary Figure S3 HFs of one-year-old (1 yo) Control and Rspo3 mice during telogen state. **(A,B)** Immunofluorescence staining of 1 yo Control and Rspo3GOF mice targeting the proliferation marker—Ki67 **(A)** and the cell apoptosis marker—Caspase-3 **(B)**. DAPI counterstaining was used to label cell nuclei. Scale bar, 20 μm.

Supplementary Figure S4 Differentiation of keratinocytes isolated from Rspo3GOF and Control mice upon increasing calcium concentration (0.05 mM ^Ca2+^, 0.3 mM ^Ca2+^, 0.8 mM Ca^2+^). Immunofluorescent staining targeting the keratinocyte differentiation markers: Keratin10 **(A)** Keratin14 **(B)**, and Loricrin **(C)**. DAPI counterstaining was used to label cell nuclei. Scale bars, 20 μm.

Supplementary Figure S5 Rspo3GOF keratinocytes displayed decreased ability to proliferate and migrate. **(A)** Pictures taken during Colony Formation Assay (CFA) analysis at different time-points after cells seeding (24, 48, 72 h, 5, 6 days). Scale bars, 50 μm. **(B)** Analysis of cells colonies, performed during different days of CFA and showing decreased number of cells per colony created by Rspo3GOF keratinocytes compared to Control. Error bars ± SEM. Data are representative of at least three independent experiments. **(C)** Immunofluorescent staining of Control and Rspo3GOF keratinocytes, targeting the cel proliferation marker – Ki67. DAPI counterstaining was used to label cell nuclei. Scale bars, 50 μm. **(D)** Rspo3GOF keratinocytes had decreased ability to proliferate compared to Control. Error bars ± SEM. Data are representative of at least three independent experiments. **(E)** Analysis of wound closure performed at different timepoints of Wound Healing test (4, 6, 24, 48 h) and showing that Rspo3GOF keratinocytes had impaired migration ability. Error bars ± SEM. Data are representative of at least three independent experiments.
