# Supplementary figures and images for: The overexpression of R-spondin 3 affects hair morphogenesis and hair development along with the formation and maturation of the hair follicle stem cells
Source: Front Physiol. 2024 Sep 16;15:1424077. doi: 10.3389/fphys.2024.1424077 (PMC11439821; doi:10.3389/fphys.2024.1424077)

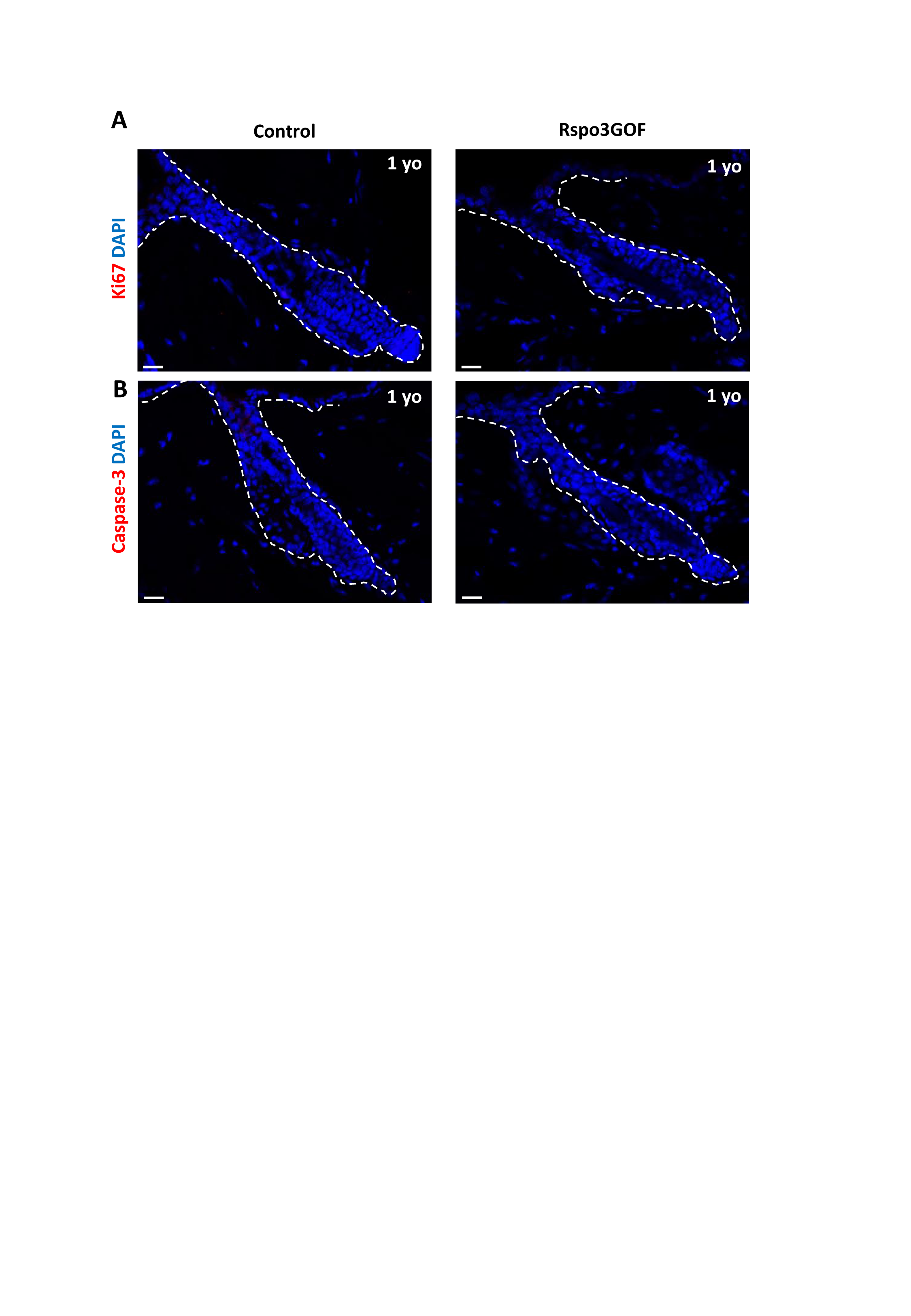

Supplement: Supplementary file 1 [file Image3.TIF]

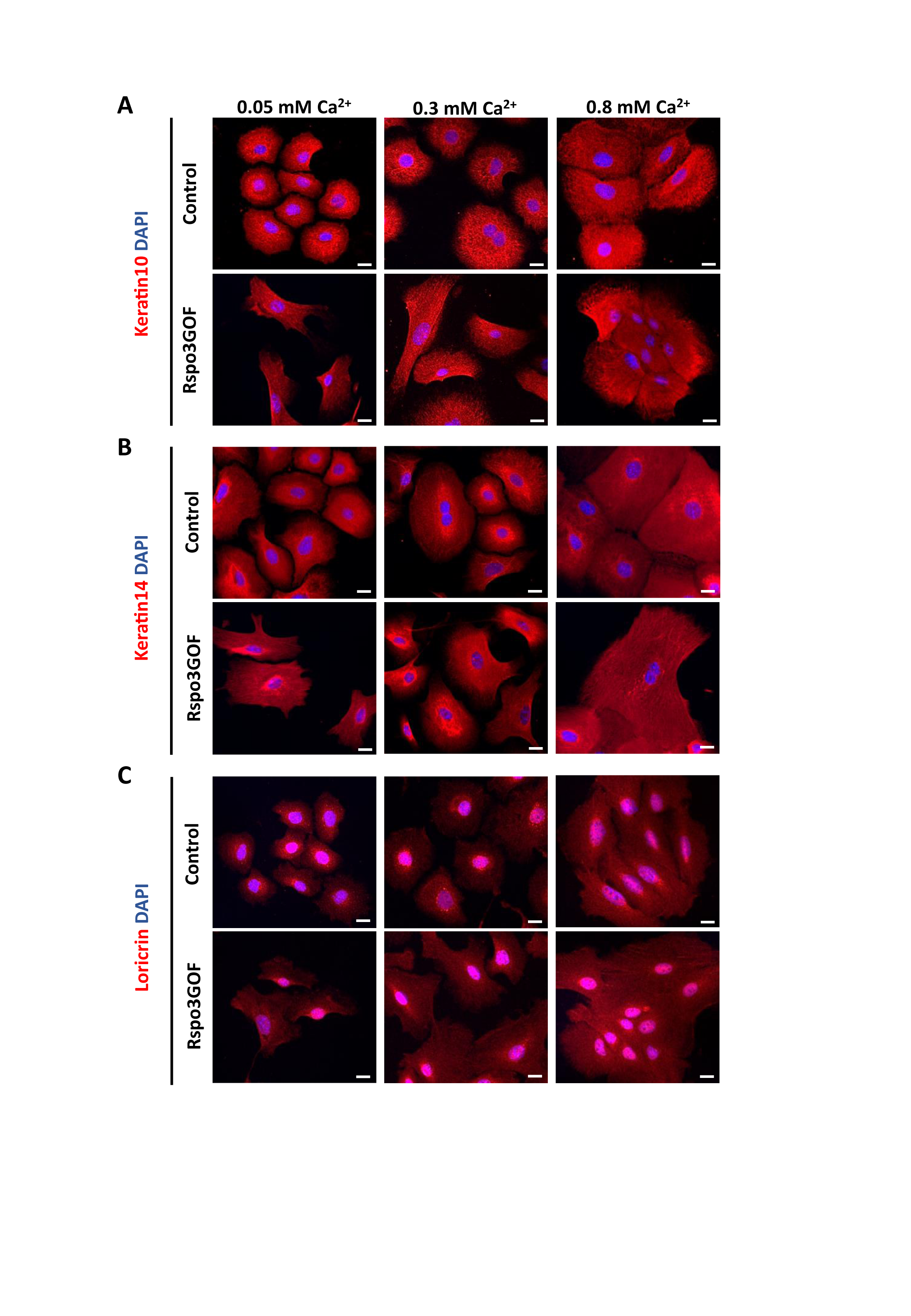

Supplement: Supplementary file 2 [file Image4.TIF]

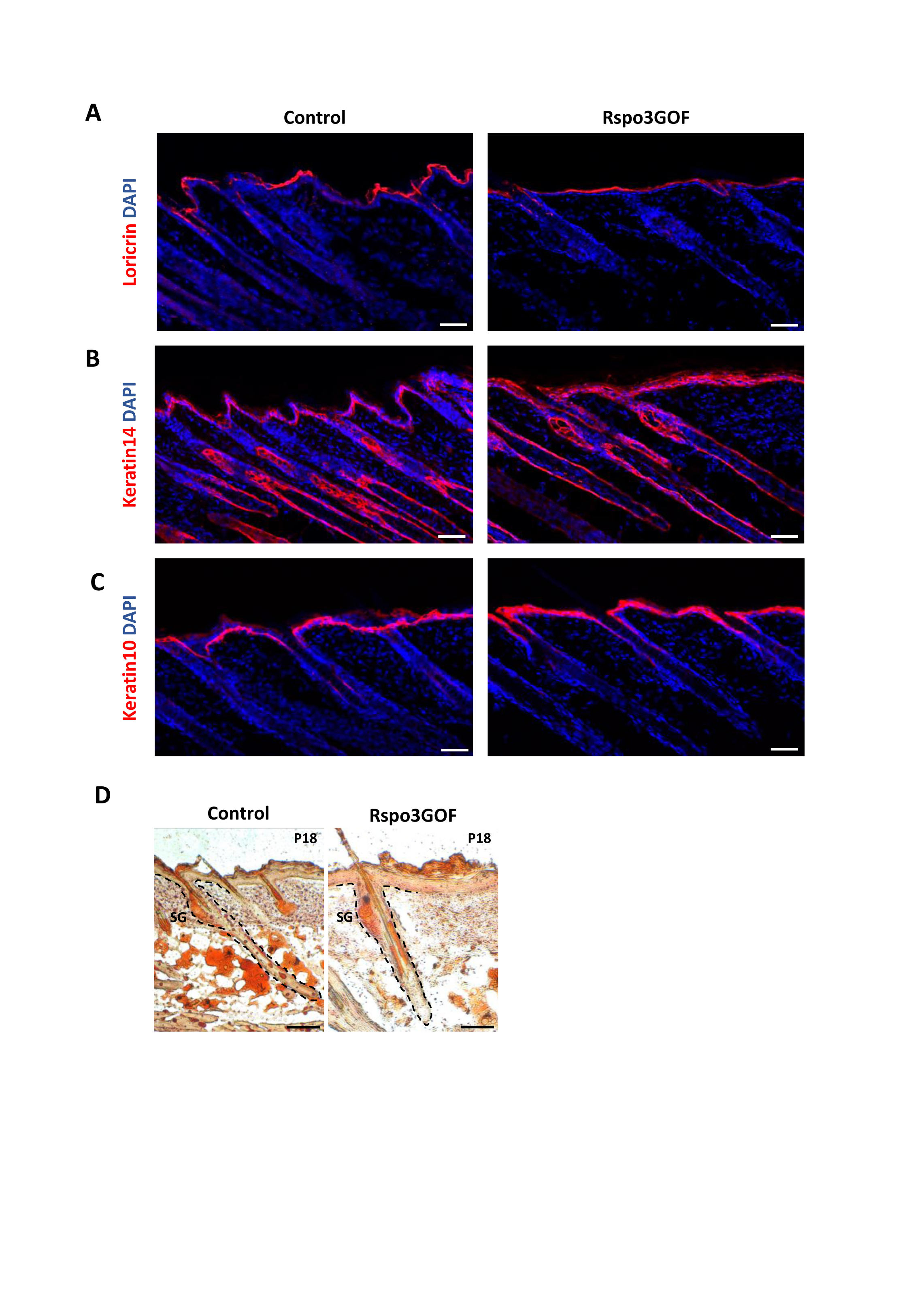

Supplement: Supplementary file 3 [file Image2.TIF]

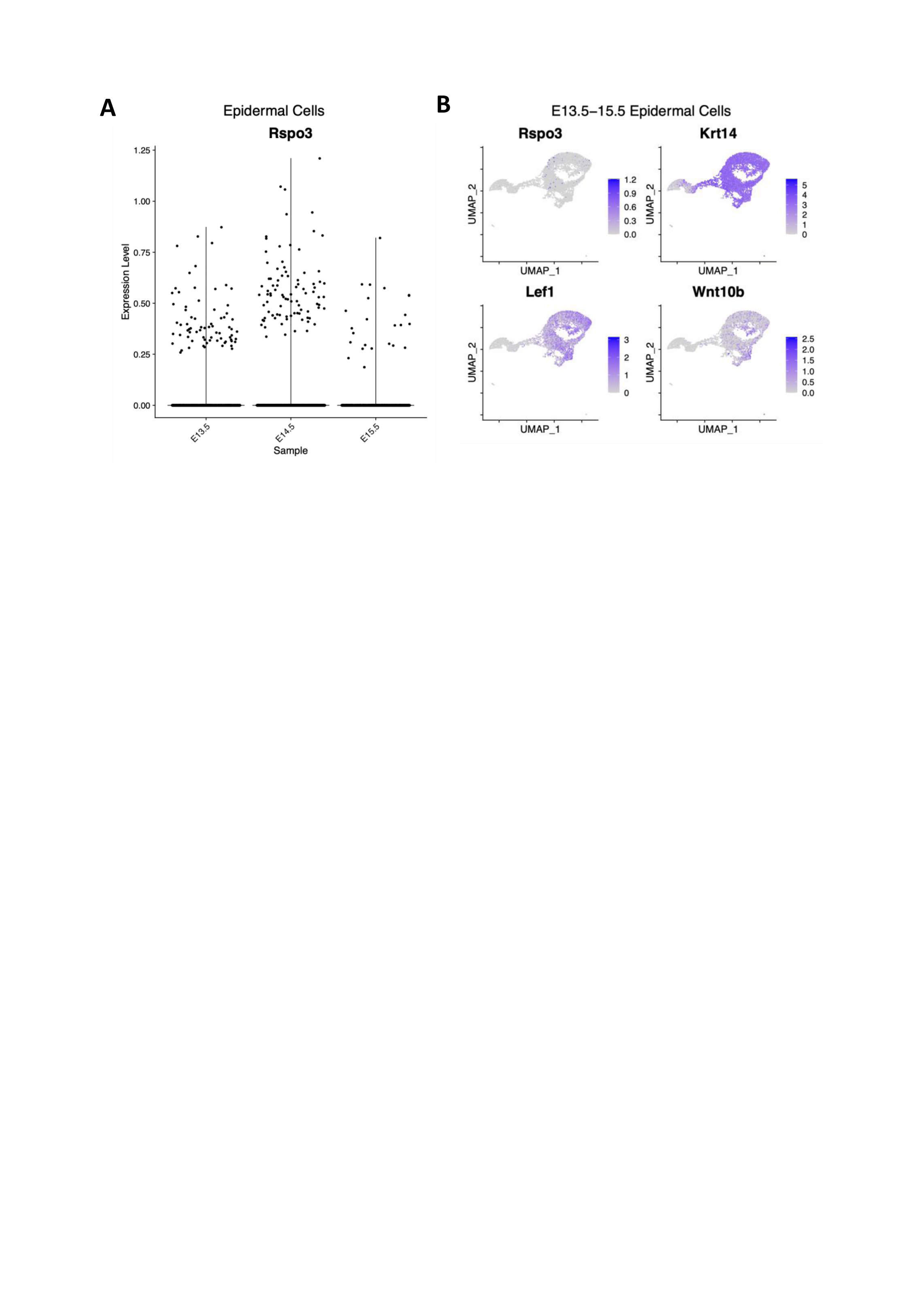

Supplement: Supplementary file 4 [file Image1.TIF]

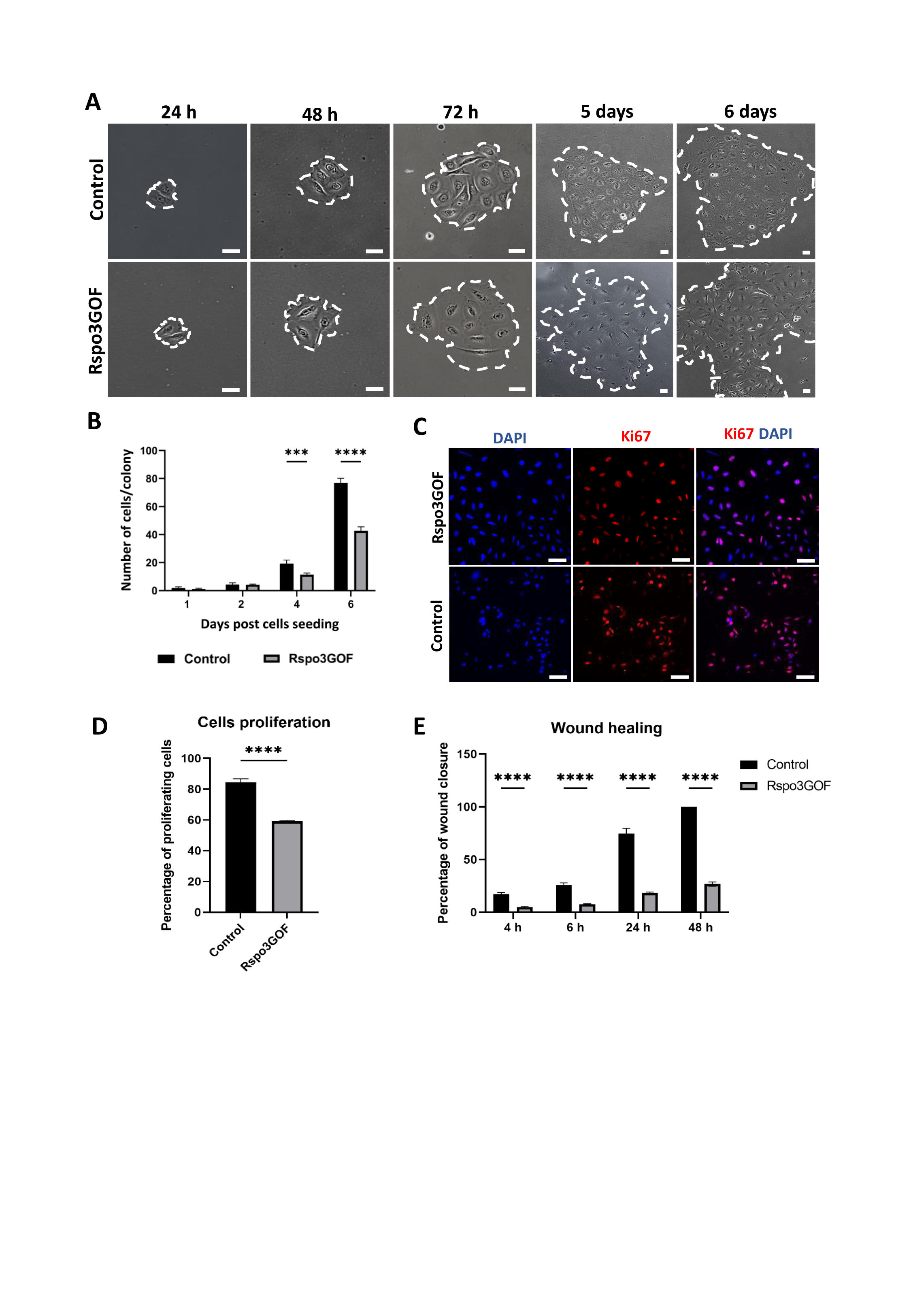

Supplement: Supplementary file 5 [file Image5.TIF]
